# Chances for learning intraprofessional collaboration between residents in hospitals

**DOI:** 10.1111/medu.14279

**Published:** 2020-08-14

**Authors:** Natasja Looman, Cornelia Fluit, Marielle van Wijngaarden, Esther de Groot, Patrick Dielissen, Dieneke van Asselt, Jacqueline de Graaf, Nynke Scherpbier‐de Haan

**Affiliations:** ^1^ Department of Primary and Community Care Radboudumc Nijmegen the Netherlands; ^2^ Department for Research in Learning and Education Radboudumc Health Academy Nijmegen the Netherlands; ^3^ Department of Geriatric Medicine Radboudumc Nijmegen the Netherlands; ^4^ Julius Center for Health Sciences and Primary Care University Medical Center Utrecht Utrecht the Netherlands; ^5^ Radboudumc Health Academy Nijmegen the Netherlands

## Abstract

**Context:**

Intraprofessional collaboration (intraPC) between primary care (PC) doctors and medical specialists (MSs) is becoming increasingly important. Patient safety issues are often related to intraPC. In order to equip doctors well for their task of providing good quality and continuity of care, intraPC needs explicit attention, starting in postgraduate training. Worldwide, PC residents undertake a hospital placement during their postgraduate training, where they work in proximity with MS residents. This placement offers the opportunity to learn intraPC. It is yet unknown whether and how residents learn intraPC and what barriers to and opportunities for exist in learning intraPC during hospital placements.

**Methods:**

We performed an ethnographic non‐participatory observational study in three emergency departments and three geriatric departments of five hospitals in the Netherlands. This was followed by 42 in‐depth interviews with the observed residents and supervisors. The observations were used to feed the questions for the in‐depth interviews. We analysed the interviews iteratively following the data collection using template analysis.

**Results:**

Hospital wards are rich in opportunities for learning intraPC. These opportunities, however, are seldom exploited for various reasons: intraPC receives limited attention when formulating placement goals, so purposeful learning of intraPC hardly takes place; residents lack awareness of the learning of intraPC; MS residents are not accustomed to searching for expertise from PC residents; PC residents adapt to the MS role and they contribute very little of their PC knowledge, and power dynamics in the hospital department negatively influence the learning of intraPC. Therefore, improvements in mindset, professional identity and power dynamics are crucial to facilitate and promote intraPC.

**Conclusions:**

Intraprofessional collaboration is not learned spontaneously during hospital placements. To benefit from the abundant opportunities to learn intraPC, adjustments to the set‐up of these placements are necessary. Learning intraPC is promoted when there is a collaborative culture, hierarchy is limited, and there is dedicated time for intraPC and support from the supervisor.

## Abstract 2

While critically important, intraprofessional collaboration is not learnt spontaneously. Improvements in mindset, professional identity and power dynamics are crucial to its promotion.

## 1 INTRODUCTION

Adverse events resulting from human error are reported frequently in health care.^1^, ^2^ A common contributing factor to these events is an ineffective collaboration between primary care (PC) doctors and medical specialists (MSs).^3^, ^4^ Frequently reported issues are deficient communication and information transfer.^4^, ^5^ These problems could increase in many health care systems because of the current tendency to translocate part of health care service provision from hospital to PC settings.^6^ This involves transitions of both patients and knowledge, leading to an increased risk of error.^3^, ^4^, ^7^ Therefore, in addition to being proficient in their professional work, PC doctors in the PC setting and MSs in the hospital should be aware of each other's context, expertise and roles, and how to communicate and collaborate intraprofessionally.^8^, ^9^

In order for doctors to be well equipped for their task of providing continuity of care between PC and the hospital setting, intraprofessional collaboration (intraPC) needs to receive special attention during postgraduate training.^10^ This can be realised by intraprofessional education (intraPE).^11^ However, the distance, both physical and conceptual, between PC and MS workplace and teaching environments seems to be a deeply rooted obstacle to this strategy.^8^ During postgraduate training, PC residents and MS residents do collaborate around referral to and discharge from the hospital. Their training programmes, however, take place in isolation from each other and focus on their own specialties.^8^ In the Netherlands, learning during PC and MS postgraduate training is predominantly workplace based. Both curricula and clinical commitments limit the time PC residents and MS residents can work together.^10^, ^11^ As a result, the opportunity to build on and learn from and about the strengths of each other is limited. Because the proximity of different professions in shared educational and clinical spaces and sufficient time allocation can help to build mutual rapport,^12^ it is precisely the proximity that requires specific attention when organising intraPE.

We explored whether and how intraPE could be organised during hospital placements. In many countries, PC residents, such as general practitioner residents and elderly care physician residents (see Box 1), undertake a hospital placement during their postgraduate training.^13^, ^14^ This hospital ward, where PC residents and MS residents work in proximity, offers the opportunity to learn intraPC through intraPE. Currently, formalised intraPE is limited; therefore, if learning of intraPC occurs, it will be predominantly unintentional.^8^ To the best of our knowledge, there has not been investigation of whether and how PC residents and MS residents learn intraPC during these placements.

Box 1 **Definitions of professionals and settings within the Dutch health care system**

**Table:** 

General practitioner (GP)	Doctor ‘working in the frontline of a healthcare system, taking the initial steps to provide care for any health problem(s) that patients may have […] including prevention, diagnosis, cure, care, and palliation’^15^
Elderly care physician (ECP)	Doctor working in long‐term care for elderly people and chronic patients, mostly in a nursing home. In the Netherlands this is a PC specialty^16^, ^17^
Primary care (PC)‐setting	The first, community‐based medical care. The PC doctors are (amongst others) GPs or ECPs. The gatekeeping role of PC doctors makes them responsible for adequate referral of patients to hospital care
Primary care resident (PC resident)	In this study, PC residents are GP residents and ECP residents. The postgraduate PC training involves a 3‐year competency‐based programme.^18^ The PC residents provide patient care in the PC setting (first and third year). During their second year, PC residents undertake other placements, amongst which is a hospital (6‐9 months)^17^
Medical specialist (MS)	Doctor providing specialist medical care. Mostly offered in hospital settings, where both inpatient and outpatient clinics are combined
Medical specialist resident (MS resident)	Doctor in training for MS. The postgraduate MS training involves a 4‐6‐year competency‐based programme. The MS residents provide patient care in a hospital

This study aims to gain insight into the potential of hospital placements for learning intraPC, by answering the following questions: (a) When and how do PC residents and MS residents learn intraPC during hospital placements?, and (b) What are opportunities for and barriers to learning intraPC during these placements?

## 2 METHODS

We carried out a constructivist ethnographic study. A constructivist approach acknowledges that researchers’ background assumptions, disciplinary perspectives and programmatic efforts along a line of study shape their research processes and conceptual emphases. Therefore, in our study, particular time and attention were paid to reflexivity throughout the research process on how our assumptions and perspectives have shaped our data collection and interpretation. The research group consisted of general practitioners, educational scientists, a psychologist, an internist, a geriatrician and a medical student. All group members were experienced in providing intraPE and/or conducting research into intraPE in different contexts. This multidisciplinary research group functioned as a form of triangulation as it brought together disciplines whose profession or training calls on highly different assumptions and knowledge areas.^19^, ^20^ An experienced psychologist (NL) and a medical student (MvW) performed the observations and interviews. Both researchers were trained in qualitative methods and analysis. For the ethnographic research, these researchers were trained during this study by an anthropologist and an educational science researcher.

### 2.1 Rapid ethnography

We used a non‐participatory rapid ethnographic research approach.^21^ Particularly in health care and medical education research, ethnographic approaches have been considered appropriate to study professional groups, sociocultural aspects and the organisation of health care and medical education.^22^, ^23^ Lingard et al^24^ describe how ethnographic research is well suited for capturing the complexity of the daily practice of interprofessional education and collaboration. Compared to classic ethnographic research, which focuses on understanding a cultural phenomenon, a rapid ethnographic research approach prescribes that researchers enter the field with a more well‐defined and focused research question and scope.^21^, ^23^, ^25^, ^26^ Rapid ethnography‐based methods provide a means of collecting data within a short, well‐defined timeline by using triangulation of observations, in‐depth interviews and theory.^21^ In this study, we collected data by observations within daily practice and in‐depth interviews to gain insight into what is already being done and to explore opportunities for and barriers to learning intraPC between PC and MS residents within hospital placements.

### 2.2 Study setting and inclusion

Using purposeful sampling techniques, we sampled emergency departments and geriatric departments of both academic and regional hospitals in the Netherlands. After inclusion, we announced our visit with posters and emailed an information letter explaining the purpose of our study, including an invitation for the interview to all residents and supervisors. For the interviews, we applied purposive sampling, including snowballing. We sampled younger and older residents and supervisors and we talked about the results with participants. This allowed us to gather broad and deep information on learning intraPC during hospital placements. We excluded residents and supervisors who worked in the hospital department for less than 1 month.

### 2.3 Data collection

Data collection through observations and in‐depth interviews was piloted by one researcher (NL). Two researchers (NL and MvW) then performed the observations and in‐depth interviews. Prior to our visit, we agreed with the supervisor, which moments would be observed. Work‐related activities and settings with potential intraPE moments were observed, for example, educational sessions, team meetings, mutual consultations and daily administrative work practice. Both researchers (NL and MvW) were familiar with the context of hospital placements.^23^ They immersed themselves in the flow of events, including informal conversations. We only performed observations at locations where no patients were involved. To improve internal reliability, the researchers (NL and MvW) carried out the first two observations and in‐depth interview together and determined and discussed differences. During the observations, the researchers (NL and MvW) made handwritten fieldnotes, which were transcribed the same day. During short observations, we produced short and direct reports instead of a thick traditional description.^21^ The fieldnotes and reports were transformed into descriptive notes and were used to inform the interview questions. This means that a different set of questions have been asked of all participants. After the interviews, all field data were anonymised.

The interviews were semi‐structured; the interview script (Appendix S2) was designed by four investigators (NL, MvW, CF and NS‐dH) The interviews were performed after a couple of observations, to ensure that the researchers had enough time to read the fieldnotes and formulate additional questions. The interviews were all conducted in person: 39 in a private room at the hospital department and three by phone. Participants were compensated with a gift card (value €20). All interviews were recorded, anonymised and transcribed verbatim.

### 2.4 Data analysis

Transcripts of the interviews were analysed using a template analysis method.^27^ We chose template analysis as in this way we could handle the large dataset more comfortably than some other methods of qualitative data.^28^ The use of a priori themes within template analysis helps focusing on themes that need to be incorporated into the analysis. A first template was developed by NL and MvW. The codes of this preliminary template were derived from the main questions from our interview guide but also arose from inspection of the data.^27^ After each day of observations and interviews, the researchers (NL and MvW) discussed their findings. The first three interviews were coded by two researchers (NL and MvW), leading to an initial coding template. After re‐reading our data and discussing our template we decided to use this template as this would represent the data as fully as possible. It contained higher level codes (representing major themes) and low to lower‐level codes, representing more specific topics. The next 39 interviews were analysed with members of our multidisciplinary coding team, consisting of NL, MvW, MV, CF, NS‐dH and EdG in various combinations. NL coded and analysed all interviews to provide continuity. Finally, all 42 transcripts were double‐coded by members of the research team (NL, CF, MvW, EdG, PD, DvA, JdG and NS‐dH).

The vast quantity of data, comprising 45 hours of observation and 42 interviews, made analysing data and finding patterns complex.^25^ Due to a clear distinction between three different groups (supervisors, PC residents and MS residents) and the use of a template analysis method in a large research team, we were able to properly analyse the large dataset.^28^

The research group (NL, MvW, NS‐dH, CF, EdG and JdG) discussed the data iteratively; all inconsistencies in applications of the codebook were discussed and resolved through consensus. Based on the discussions, NL adjusted the template. During the coding process NL discussed the results with CF, who is a researcher in the field of workplace learning. These discussions helped challenge NL’s interpretation of the data and introduce alternative interpretations. After analysing the interviews, the fieldnotes of the observations were re‐read to check for discrepancies between fieldnotes and interview data. The number of observations and interviews was determined by theoretical sufficiency.^29^ Data collection was finished when the research group concluded they had reached ‘meaning saturation’^30^ and conceptual depth to answer the research question.^29^

### 2.5 Ethics

This study was reviewed and approved by the NVMO (Netherlands Association for Medical Education) Ethical Review Board (NERB dossier number 983). Written informed consent of all participants was obtained before participation. In some cases, nurses, (para)medical professionals and medical students were visible during the observations and therefore they were asked for informed consent to be observed, after receiving an information letter on the day of our observations.

## 3 RESULTS

We conducted 45 hours of observations (10‐360 minutes per observation) and 42 interviews (18‐50 minutes per interview) with 14 PC residents, 14 MS residents and 14 supervisors at three emergency departments and three geriatrics departments of five hospitals from February to May 2018 (Table 1 and Appendix S1).

**TABLE 1:** Participants for observations and interviews

Participant	Total	Male	Female	Age (range)	Year of specialty training
Primary care residents	14	5	9	32.2 (28‐50)	
GP residents	11	4	7		2
ECP residents	3	1	2		2
Medical specialist residents	14	5	9	30.5 (26‐37)	
ER residents	6	2	4		1, 1, 1, 3, 3, 3
Geriatric residents	5	1	4		3, 3, 5, 5, 5
Surgery resident	1	1	0		1
Internal residents	1	0	1		5
Hospitalist^a^ resident	1	1	0		1
Supervisors (medical specialists)	14	7	7	49.6 (34‐64)	9.6 years (1‐18) supervising experience

^a^ New specialisation in the Netherlands for generalist doctors within the hospital. Abbreviations: ER, emergency care; ECP, elderly care physician; GP, general practitioner.

A prevailing view amongst all PC and MS residents and supervisors was that intraPE is essential and needs explicit attention, including dedicated time.

*To me, it [intraPE] is super important, and it should receive more attention.* (MS‐resident1_H3)

All participants indicated that hospital wards are rich in opportunities to learn intraPC. To actually benefit from these opportunities, interventions are needed. After categorisation of the results, we identified three main themes: (a) incidental and purposeful learning; (b) competing professional roles, and (c) work environment. In relation to these three themes, residents and supervisors mentioned clear recommendations for the introduction and implementation of intraPE during hospital placements.

### 3.1 Theme 1. Incidental and purposeful learning

Our data showed that learning intraPC on the hospital ward occurs by two routes: (a) incidental (implicit learning activities), and (b) purposeful (explicit learning activities).

### 3.2 Learning implicitly and incidentally

The majority of intraprofessional learning activities occurred implicitely during daily work activities. Though working, PC residents learned about the daily hospital routine: substantive medical skills; how to best refer a patient to the hospital, and how to formulate an adequate referral question. The PC residents usually learned these skills from the supervisors in the hospital department. Supervisors also operated as a role model in intraPC; MS residents often copy their behaviour. The MS residents learned about possibilities and limitations in the PC setting and referral patterns, mostly from PC residents. Residents mentioned that the learning of intraPC mainly occurred incidentally, without conscious reflection.

*Some attention is paid to intraPC, but it is not really high on the agenda. You do notice that they know PC‐residents are walking around and sometimes get questions for example ‘is that possible in the nursing home?’ or ‘how do you see that as GP?’ or ‘How would you feel if we discharge such a patient?’ This kind of interaction happens spontaneously. (PC‐resident1_H2)*

### 3.3 Learning explicitly and purposefully

We observed that intraPE is purposeful and planned in some departments, especially in departments with a collaborative culture, dedicated time for intraPE and intraPE mindset of the supervisor (see Box 2).

Box 2 **Example of purposeful and planned intraPE**

**Table:** 

A joint intraprofessional team reflection followed directly after the weekly intraprofessional grand round at the geriatrics department. This form of intraprofessional education (intraPE) occurs every Wednesday from 09.00 AM to 10.00 AM
During the grand round today, 11 participants are participating: supervisors, medical specialist (MS)‐resident, primary care (PC)‐resident (general practitioner [GP‐resident] and elderly care physician [ECP‐resident]), and medical students. Each patient is seen by a PC‐resident and a MS‐resident together (in various combinations), the other participants are observing this intraprofessional consultation. After this grand round, a joint team discussion and reflection takes place in the handover room. Everyone is seated around the table. One of the PC‐residents (GP‐resident) presents a patient, followed by discussion between the two PC‐residents, supervisor 1 and supervisor 3. Supervisor 2 is observing and supervisor 4 occasionally asks questions during this discussion. When the discussion is about medication for the patient, the GP‐resident asks: *‘is this the regular medication for this type of complaints (problem behaviour)?’* The ECP‐resident shakes his head *‘no’*. Supervisor 1 invites this ECP‐resident to explain the elderly care guidelines of problem behaviour. The ECP‐resident explains the updated guidelines for problem behaviour (used in primary care). Supervisor 1 says: *‘thus, we cannot provide medication for the treatment of problem behaviour, due to lack of evidence for the effect of medication on problem behaviour.’* Supervisor 4 asks the other residents and medical students: *‘can you follow our thoughts?’* The supervisors invite everyone to ask questions and to share their expertise. Note observer: The atmosphere is relaxed, there seems to be enough time and space for questions and education, residents and students are invited to share knowledge and to ask critical questions. There is room for one's own opinion and disagreeing with each other, the atmosphere remains relaxed and respectful. Fieldnote_R1_H2.

### 3.4 Role of supervisor in purposeful learning

The PC residents indicated that some supervisors consciously stimulate interaction between PC residents and MS residents and encourage PC residents to show their PC expertise.

*I always try to share my PC‐vision […] It is a positive thing, that they really appreciate it that I have a vision as a general practitioner. And I also get to hear that they [supervisors] really like it that I contribute my PC‐opinion. That is of course stimulating*. (PC‐resident1_H5).

Supervisors mentioned that they find it difficult to coach and assess the learning of intraPC for residents from different backgrounds. Their expertise is based on specialist medical knowledge and skills, and they feel competent in teaching in this area, but offering intraPE poses specific demands beyond their primary expertise. In order to provide intraPE, supervisors feel a need to study new knowledge and skills regarding collaboration. (Supervisor2_H2)

*What I mention about just [the use of] theories about collaboration, we haven't done it before, but lately occasionally. Yes as a doctor, you know very little about that, we just do it. Sometimes it goes well and sometimes it doesn't work. Things like that are, I should delve into it [theory about collaboration]*. (Supervisor2_H2).

### 3.5 Placement goals

Both PC residents and MS residents indicated that learning intraPC is essential, but they are not always aware of opportunities to learn this. Residents are accustomed to operating and learning separated from each other and indicate that intraPE is generally not in their mindset.

*I can ask her [PC‐resident] ‘how are things organised in your PC‐setting?’ We do discuss such things, and she tells me a lot about that […] But really learning to collaborate, no. We are each on our own island, and you occasionally ask something about ‘how are things going on your island.* ‘ (MS‐resident1_H2)

The MS residents often teach PC residents about their medical specialty and how the hospital is organised, but they hardly ever ask for PC expertise, with the result that PC residents think that MS residents do not want to learn from them. This means that learning is predominantly unidirectional.

*Of course I learn a lot from his or her [MS‐resident] knowledge and skills […] Conversely I have the idea that they learn less from us. And that they do not really want it either. Then it is a bit of one‐way learning.* (PCߚresident1_H5)

Residents and supervisors reported that intraPC receives at best limited attention in the training programmes as a competency to be learned during hospital placements. Therefore, the learning predominantly depends on residents’ individual mindsets on learning intraPC to formulate learning goals within this domain. Some supervisors would like PC residents to be obliged to formulate a learning goal for intraPC, but supervisors indicated that they never insisted that MS residents should formulate such a learning goal.

*PC‐residents have different [training] goals and portfolios than MS‐residents. And they [PC‐residents] steer very much on their own learning objectives. So then you might have to make a standard learning goal for them. That you say that the collaboration between PC‐residents and MS‐residents is one of the learning goals for all PC‐residents who come here.* (Supervisor2_H6)

The PC residents expected attention would be given to intraPC during release days, where PC residents learn amongst their peers at the PC specialty training institute once a week, but they indicated that intraPC receives only limited attention.

*[Question: Are learning intraPC and sharing PC expertise themes during release days?] Very few actually and that surprised me. I thought that there would surely be attention (for intraPC), also for the collaboration with the elderly care physician. But that [intraPC] is actually not discussed at all.* (PC‐resident2_H6)

### 3.6 Theme 2. Competing professional roles

The observations and interviews showed that PC residents often adjust to the role of MS resident, providing specialist medical care during their hospital placement as if they are in training for that MS specialty. The majority of PC residents hardly ever share their PC knowledge and skills, except when invited.

*The PC‐resident steps into our MS‐role and that is also what is [implicitly] expected. It is a fact that they act just as MS‐residents; they have to drop their PC‐role to say the least*. (MS‐resident1_H6)

*During handovers, when we discuss the patient's discharge we don't know if a GP can do anything with our suggestions. Then, we should explicitly invite PC‐residents to say how their view is; they don't do that on their own*. (Supervisor2_H3)

The PC residents, who continued to adapt to the role of MS resident and barely expressed their professional PC identity, sometimes even were not aware of their PC knowledge and skills.

*And he [MS‐supervisor] told me ‘you have to ask hard questions to the other disciplines like how far will we go in our decisions?’ And then I thought, of course, that is actually something I normally do in primary care. Well, I won't say that I really forgot it, but I think that I was too much in the MS‐role.* (PC‐resident2_H6)

The PC residents, who easily switched between the MS role and their professional PC identity, were more explicit and proactive in demonstrating their PC expertise.

*I also give some kind of information back to the specialists which they can use. I see myself more as a general practitioner within the ER I know something about emergency cases, and I also know a lot about general practice. With that, I can also put them [MSs/MS‐residents] in the right direction.* (PC‐resident1_H4)

### 3.7 Theme 3. Work environment

A prevailing view amongst participants was that learning intraPC between residents is only possible when a safe work‐learning climate and significant practicalities are secured.

### 3.8 Work‐learning climate

We observed that the placement of residents within the room during team meetings can reflect (in)equality. Within some departments, everybody was seated equally in the room. In other departments, PC residents were not sitting around the table amongst other MS residents, but sitting or standing in the second rank. The discussion took place at the table and the second rank (PC residents) were acting as spectators. The placement of residents was affecting the chances for intraPE.

*The medical specialists are sitting at the head of the U‐form table, and the MS‐residents are sitting on the sides of the table. The PC‐resident is sitting on the second row, together with undergraduate students. The PC‐resident is the only doctor who have to take place second rank between the students.* (Fieldnote_R2_H4)

Another essential aspect to creating a safe work‐learning climate is ‘knowing each other,’ for example, by having a drink together outside the ward. Residents and supervisors who know each other informally report that they get in touch with one another more easily, understand why people react the way they do and are more likely to invest in each other. Participants mentioned that hierarchy, such as that between supervisor and resident, is useful as it clarifies roles and responsibilities within the hospital. Nevertheless, they indicated that too much power dynamics in the hospital ward can lead to a lack of respect and inequality, which has a hindering effect on building a relationship in order to get to know each other's expertise. Supervisors and residents mentioned that the way MS speak about PC doctors can be responsible for creating a (un)safe work‐learning climate for intraPE.

*Sometimes medical specialists talk about primary care doctors in a negative way, like it's an inferior specialism. And sometimes I hear such comments during meetings between shifts, that is of course demotivating.* (PC‐resident3_H1)

Participants mentioned that supervisors are in the position to steer power dynamics, and within some departments supervisors showed an active policy against unconstructive power dynamics.

### 3.9 Practicalities

Intraprofessional education can hardly take place when PC and MS residents are working in different shifts or having different offices. Supervisors and residents indicated that the opportunity to meet each other is necessary for intraPE to take place. This is possible by sharing physical space together.

*We are in a set‐up in which we sit in a circle (behind computers) and where you easily pick up things from each other. And then an interesting (intraprofessional) discussion, a case‐based discussion arises spontaneously.* (Supervisor2_H5)

### 3.10 Residents’ and supervisors’ perceived needs

In relation to the above themes, residents and supervisors mentioned clear recommendations to identify the different workplace activities for learning of intraPC and to explicitly integrate workplace opportunities: (a) the specific organisation of work context by creating actual possibilities for learning intraPC; (b) explicit and purposeful learning of intraPC during workplace activities, by both PC residents and MS residents; (c) supervisors taking responsibility for intraPE by facilitating a constructive work‐learning climate and further professional development in intraPE; (d) intraPC as a placement goal for both PC residents and MS residents; (e) empowerment of PC residents to share their PC expertise; (f) empowerment of MS residents to ask for PC expertise, and (g) offering placements for MS residents in the PC setting.

*It [intraPE] must be integrated in the placement/work structure. When it is something optional or incidentally, then it will not work out*. (Supervisor2_H3)

*For example case‐based discussions, where we discuss the kind of cases that we all recognize. And that we also hear their [PC‐residents’] side of the story and also hear from them what they encounter when collaborating with us, and vice versa. I think that is very important*. (MS‐resident1_H6)

*For us [MS‐residents], a placement in a nursing home would also be a very good idea. That is not at all in our training program […] It can sometimes be quite difficult if you have no idea at all about how it works in a nursing home […] I think it is very good that we make that more transparent and learn from each other.* (MS‐resident1_H3)

*I think that we will only get a real collaborative relationship if they [MS‐residents] also come along when I am in the primary care practice*. (PC‐resident1_H6)

## 4 DISCUSSION

All participants found intraPC essential for good health care and consider hospital wards to be rich in opportunities for learning intraPC. However, we also report that these opportunities are seldom exploited for various reasons. First, intraPC learning goals are often not apparent and both residents and supervisors lack awareness of the intraPC learning opportunities. When learning intraPC occurs, it is predominantly implicit. Second, PC residents often adapt to the role of MS resident and hardly ever share their PC expertise. The MS residents often neglect to search for PC expertise. Third, too much hierarchy led to inequity, which had a hindering effect on building relationships and formed a not‐safe‐enough work‐learning climate in which residents did not feel free to speak up. Therefore, improvements in mindset, professional identity and power dynamics are crucial to facilitate and promote intraPC.

### 4.1 Mindset

When learning of intraPC occurs between PC residents and MS residents, this is mostly random through informal mechanisms: the learning occurs implicitly, spontaneously and with little conscious reflection, which is in line with the description by Watkins and Marsick of informal and incidental learning.^31^ To our knowledge, our study is the first to investigate intraPE during hospital placements. Our findings are consistent with previous studies in other contexts, which also showed that learning of collaborative competences lacks structured implementation and is generally not in the mindset of medical professionals.^11^, ^32^, ^33^ Residents are expected to learn during their postgraduate training and, therefore, it could be expected that they are always on the lookout for learning opportunities. However, with regard to intraPC, this happens only to a limited extent.

Frequently, mindset is associated with the growth mindset theory from Dweck.^34^ However, in social psychology and organisational leadership, mindset is seen as a cognitive filter through which one looks at the world, a predefined reference frame, ‘used throughout the totality of an individual or organization's cognition.’^34^ Johnston clearly recasts a long‐standing idea when she states that ‘excellent medical education occurs in secondary care settings’^7^ and elaborates that primary care has an ‘inferior status’^7^ and is considered to be much less advanced. Consequently, MS residents teach PC residents, but they are not accustomed to asking for PC expertise from PC residents, maybe not realising or appreciating their PC expertise. The MS residents rarely have placements in PC settings. These historical patterns can lead to a mindset for predominantly unidirectional learning at the workplace. Uhlig et al described that, in order to successfully realise interprofessional collaboration, many deeply rooted patterns, role cultures and assumptions must be carefully adjusted.^35^ Our results underscore that MS supervisors and PC teachers have an important role in creating a mindset for learning intraPC. They can do this by formulating placement goals for both PC residents and MS residents and by stimulating two‐way learning and conscious reflection.^36^, ^37^

The above indicates that intraPE is the responsibility of all parties involved: PC residents; MS residents; supervisors; teachers, and programme directors.

### 4.2 Professional identity

In the Netherlands, the purpose of hospital placements for PC residents is to gain expertise in emergency care and diseases that are not very prevalent in a PC setting and to learn intraPC with medical specialists. We found that PC residents often adapt to the role of MS resident. This is useful for learning medical skills and fitting into the hospital team. However, the majority of PC residents hardly ever share their PC expertise. This is counterproductive for learning intraPC. At first glance, the PC resident appears to have little influence on the dynamics of an expert team within the hospital ward. However, our results show that also temporary team members can bring a fresh eye to common practices. We found that PC residents who expressed their professional PC identity and easily alternated between the MS role and PC role, created intraPC discussions and bidirectional learning. Previous literature shows that pre‐existing teams are more receptive to the influence of newcomers when the newcomers are more assertive.^38^ Proactive PC and MS residents would also rapidly take charge of their intraPC learning process once they are included in the learning cycle.^31^ This stresses the importance of empowering PC residents to express their professional identity and to proactively share their PC knowledge, and empowering MS residents to proactively ask for PC knowledge.

### 4.3 Power dynamics

The participants mentioned that hierarchy is useful for clarifying roles and responsibilities within the hospital, but too much hierarchy can create inequity. Power is enhanced through the hierarchies in which residents interact.^39^ Hierarchy or power dynamics are barely investigated within intraPE^40^; only Meijer et al mentioned hierarchy.^11^ In their study, hierarchy did not seem to influence intraPE, which is contrary to our findings. This discrepancy may be attributed to the fact that their residents only interacted by telephone and letter; power dynamics may be less prevalent during telephone and letter interactions. Studies on hierarchy and power dynamics within in*ter*PE confirm our findings.^41^, ^42^ Baker warned that attention should be paid to factors causing hierarchy; otherwise, in‘ter’PE can increase competition and unequal power relationships (power dynamics) between professionals, which has a reverse effect on collaboration.^41^ Edmondson demonstrated that in working teams, learning behaviour, such as sharing perspectives, asking questions and seeking feedback, is highly dependent on team psychological safety: ‘a shared belief that the team is safe for interpersonal risk‐taking.’^43^ Power dynamics can have a corrosive effect on psychological safety^44^ and therefore on learning intraPC between residents. Meanwhile, informal relations are related to psychological safety.^44^ We found strong evidence that learning intraPC between residents is influenced by the degree of equity and informal relations in the hospital department. This has been identified in earlier studies as well.^11^, ^32^ Janssen et al^32^ showed that interaction between residents and supervisors, in which they take each other seriously, is a crucial factor in intraPE. Meijer et al^11^ concluded that knowing each other makes intraPC between general practitioner residents and MS residents much easier. Our study shows that equity and informal relations are promoted by practical issues such as sharing physical space, sitting equally in the room around the table, dedicated time together, having a drink together outside the workplace and speaking respectfully about each other.

### 4.4 Strengths and limitations

A strength of this study is the use of four types of triangulation: (a) method; (b) data source; (c) investigator, and (d) research group triangulation.^45^ An interprofessional research group brought together disciplines with highly diverse assumptions and different knowledge bases,^19^, ^20^ and triangulation allowed researchers to examine different data sources to confirm and contrast findings.^45^ The psychologist, for example, had a keen eye for the possible effects of adjusting hierarchy and the general practitioner focused on elaborating the importance of sharing PC expertise.

We consider the variability in the nature of the observations as a strength. The short observations consisted of five meetings lasting less than 15 minutes. These were meetings to start the day in an interprofessional way. Although short, these meetings provided us with very rich observations with respect to (opportunities for) intraPE. Because our observers were familiar with the context of hospital placements they could easily recognise relevant activities. Another strength is the cooperative attitude of residents and supervisors in participating in this study; we had to cancel some hospitals, which had applied to participate, after conceptual depth was reached. Because of this cooperative attitude, we could get a rich conception of the potential of hospital placements for learning intraPC.

We acknowledge several limitations. Our presence during observations may have had an impact on the participant reactivity, which is defined by Paradis and Sutkin as: ‘a form of participant effect that comes from participants’ active engagement with the research and its aims, leading to behavioral adaptation that aligns with perceived social norms.‘^46^ We think we minimised participants’ reactivity by being embedded in the environment and checking our observations during the in‐depth interviews with the participants.^23^, ^46^ Observers were dressed in a hospital uniform and we undertook at least four observations in every hospital department. We noticed that people did interact with us as if we were new colleagues and continued their actions seemingly uninterrupted, especially when we revisited departments. Another limitation is that we only performed observations in locations where no patients were involved. Therefore, a part of informal learning intraPC remained outside the scope of our study. By practising reflexivity in an interprofessional research group, we think this limitation was reduced as much as possible.

### 4.5 Implications for practice and future research

When organising the learning of intraPC through placements for residents from different medical backgrounds, we think the following should be kept in mind. First, informal learning can be planned or unplanned, but it involves at least some conscious reflection.^31^ It is necessary to implement intraPE within workplace‐based learning, to make the learning of intraPC purposeful. Second, the hierarchy must be taken into account; for example, by sharing a room and sitting equally around the table, asking for different perspectives, and letting PC and MS residents speak first during discussions and then letting supervisors add their information. Third, supervisors need extra training to be aware of and create learning opportunities and to create a mindset for learning intraPC. Finally, residents need some level of professional identity to be able to show their expertise and for supervisors to steer intraPE. A professional role identity is developed from a combination of personal factors, the working environment and role modelling.^47^, ^48^, ^49^ However, PC role models are absent during hospital placements. Therefore, peer‐to‐peer meetings during placements could be a valuable alternative.^49^ We recommend release days, where PC residents learn about having a dialogue with their peers about intraPC. Future research is needed to investigate how the development of professional role identity can be supported, and how power dynamics can be managed in a constructive way.

## 5 CONCLUSIONS

All residents and supervisors indicated that learning intraPC is essential and requires more explicit attention. IntraPC is not learned spontaneously during hospital placements. Even in a promising setting where PC residents and MS residents work together in the same department, intraPC receives at best limited attention as a competency to be learned. The MS residents are not accustomed to asking for PC expertise and PC residents often adapt to the role of MS resident and they hardly ever contribute their PC knowledge. Hierarchy and a lack of psychological safety in the hospital department negatively influence the learning of intraPC. We conclude that in order to benefit from the opportunities to learn intraPC during hospital placements, attention to mindset, professional identity and power dynamics is needed. Learning intraPC is promoted when there is a collaborative culture (with not too much hierarchy), dedicated time and goal setting for intraPC and support from the MS supervisor on the ward and PC teachers during release days.

## AUTHOR CONTRIBUTIONS

All authors (NL, CF, MvW, EdG, PD, DvA, JdG and NS‐dH) met all of the following conditions: substantial contributions to the conception and design of the work; the acquisition, analysis and interpretation of data for the work; drafting the work and revising it critically for important intellectual content; final approval of the submitted paper and the version to be published; and agreed to be accountable for all aspects of the work in ensuring that questions related to the accuracy and integrity of any part of the work are appropriately investigated and resolved.

## CONFLICTS OF INTEREST

There are no competing interests.

## ETHICAL APPROVAL

We asked the NVMO (Netherlands Association for Medical Education) Ethical Review Board for ethical review of our research proposal on 15 December 2017. The NVMO Ethical Review Board approved the study on 15 February 2018, dossier number 983.

## Supporting information

Appendix S1

Appendix S2
