# Supplementary material for: Chances for learning intraprofessional collaboration between residents in hospitals
Source: Med Educ. 2020 Aug 14;54(12):1109–19. doi: 10.1111/medu.14279 (PMC7754101; doi:10.1111/medu.14279)
Supplement: Supplementary file 1 — Appendix S1 [file MEDU-54-1109-s001.docx]

| \| Additional file 1. Hospital departments: six departments in two academic and three regional hospitals \| \| \| \| --- \| --- \| --- \| \| Hospital \| Academic \| Regional \| \| A \| Emergency department \|  \| \| A \| Geriatrics department \|  \| \| B \|  \| Geriatrics department \| \| C \|  \| Emergency department \| \| D \|  \| Emergency department \| \| E \| Geriatrics department \|  \| |
| --- | --- | --- | --- | --- | --- | --- | --- | --- | --- | --- | --- | --- | --- | --- | --- | --- | --- | --- | --- | --- | --- | --- | --- | --- |
