# Supplementary material for: Chances for learning intraprofessional collaboration between residents in hospitals
Source: Med Educ. 2020 Aug 14;54(12):1109–19. doi: 10.1111/medu.14279 (PMC7754101; doi:10.1111/medu.14279)
Supplement: Supplementary file 2 — Appendix S2 [file MEDU-54-1109-s002.docx]

| Additional file 2. Interview guide with starting questions | |
| --- | --- |
| *Domain* | *Question (example)* |
| *General* | *How do you think about learning intraprofessional collaboration during this hospital placement?* |
| *Current situation* | *Can you give an example of intraprofessional education during your current hospital placement?* |
| *Possibilities* | *Do you see possibilities for learning intraprofessional collaboration (between primary care and medical specialist residents)/within your current rotation?* |
| *Obstacles* | *What are the factors that could hinder intraprofessional education within your current internship?* |
| *Specific for a discipline* | *Primary care trainee: to which extent do you act as a medical specialist resident?* |
| *In response to the observations* | *Questions to clarify what is seen during the observation* |
| *End* | *Do you know colleagues who think differently about intraPE than you do?* |
